# Evaluation of decellularization process for developing osteogenic bovine cancellous bone scaffolds *in-vitro*

**DOI:** 10.1371/journal.pone.0283922

**Published:** 2023-04-05

**Authors:** Ali Al Qabbani, K. G. Aghila Rani, Junaidi Syarif, Sausan AlKawas, Suzina Sheikh Abdul Hamid, A. R. Samsudin, Ahmad Azlina

**Affiliations:** 1 Department of Oral & Craniofacial Health Sciences, College of Dental Medicine, University of Sharjah, Sharjah, United Arab Emirates; 2 Basic Science and Oral Biology Unit, School of Dental Sciences, Universiti Sains Malaysia, Kubang Kerian, Kelantan, Malaysia; 3 Sharjah Institute for Medical Research, University of Sharjah, Sharjah, United Arab Emirates; 4 Department of Nuclear and Mechanical Engineering, College of Engineering, University of Sharjah, Sharjah, United Arab Emirates; 5 Tissue Bank, School of Medical Sciences, Universiti Sains Malaysia, Kubang Kerian, Kelantan, Malaysia; Ohio State University, UNITED STATES

## Abstract

Current immunological issues in bone grafting regarding the transfer of xenogeneic donor bone cells into the recipient are challenging the industry to produce safer acellular natural matrices for bone regeneration. The aim of this study was to investigate the efficacy of a novel decellularization technique for producing bovine cancellous bone scaffold and compare its physicochemical, mechanical, and biological characteristics with demineralized cancellous bone scaffold in an *in-vitro* study. Cancellous bone blocks were harvested from a bovine femoral head (18–24 months old) subjected to physical cleansing and chemical defatting, and further processed in two ways. Group I was subjected to demineralization, while Group II underwent decellularization through physical, chemical, and enzymatic treatments. Both were then freeze-dried, and gamma radiated, finally producing a demineralized bovine cancellous bone (DMB) scaffold and decellularized bovine cancellous bone (DCC) scaffold. Both DMB and DCC scaffolds were subjected to histological evaluation, scanning electron microscopy/energy-dispersive X-ray spectroscopy (SEM/EDS), fourier-transform infrared spectroscopy (FTIR), quantification of lipid, collagen, and residual nucleic acid content, and mechanical testing. The osteogenic potential was investigated through the recellularization of scaffolds with human osteoblast cell seeding and examined for cell attachment, proliferation, and mineralization by Alizarin staining and gene expression. DCC produced a complete acellular extracellular matrix (ECM) with the absence of nucleic acid content, wider pores with extensive interconnectivity and partially retaining collagen fibrils. DCC demonstrated a higher cell proliferation rate, upregulation of osteogenic differentiation markers, and substantial mineralized nodules production. Our findings suggest that the decellularization technique produced an acellular DCC scaffold with minimal damage to ECM and possesses osteogenic potential through the mechanisms of osteoconduction, osteoinduction, and osteogenesis *in-vitro*.

## Introduction

Trauma, infection, tumor, and degenerative diseases of bone may lead to defects that need repair or regeneration. Bone substitutes are needed to support effective healing when a critical size bone defect occurs [1]. Traditional gold standard autografts for bone grafting are limited by the amount of bone available at the donor site, additional surgery causing increased surgical morbidity, and the cost of healthcare [2]. Bone allografts are popular, but availability is much lower due to the risk of transmission of viral infections, and high procurement costs. Synthetic bone alloplasts have largely replaced these needs with hydroxyapatite bone substitutes initially taking the lead [3]. Despite the advent of nanotechnology and 3D-tissue printing, many challenges are still associated with processing synthetic alloplastic scaffolds that could recapitulate the complex natural bone topography necessary for intricate communications with the cell microenvironment [4].

The pressing demand for biocompatible bone substitutes pushes the search toward xenogeneic tissues. Processed bovine bone graft scaffolds have been in use for more than half a century, with varying degrees of success, and their popularity remains due to unlimited availability [3–5]. Xenotransplantation is not without risks, and recent awareness of the long-term risk of transferring xenogeneic genetic material into the host has been raised [6–8].

Demineralized bone matrix from bovine sources, has received wide acceptance in dentistry over the past half-century [9]. It behaves as an osteoconductive and osteoinductive commercial biomaterial and is approved as a medical device used in bone defects with a long track record of clinical use in diverse forms [10]. Its low mechanical strength has limited its use as filler for small osseous defects in periodontal pathology and cystic cavities. However, it has also been applied in more advanced reconstructive skeletal work with the addition of growth factors and platelet-rich blood derivatives to enhance the regenerative potential [11]. The main preparatory process of the demineralized bovine bone matrix comprises acid extraction of the inorganic matrix while retaining much of the proteinaceous components native to the bone. The demineralization process retains small amounts of calcium-based solids, inorganic phosphates and some trace of residual native cell debris, which may pose a long-term unknown risk in the recipient.

One of the most promising techniques used to address residual native cell debris in tissue graft is through the decellularization process, whereby the extracellular matrix (ECM) is isolated from its native cells and genetic material to produce a natural scaffold without the risk of genetic material transplantation [12]. The decellularization technique has been popular with soft tissue biomaterials, but not much is known regarding the decellularization process for the development of natural bone graft substitutes [8–10].

In clinical practice, residual native cells in bovine bone graft scaffolds produce immunological responses that may be responsible for the long-standing, poorly understood chronic inflammatory reaction at the xenogenic grafted site [13]. While there is a pressing need to provide a cellular-free bone scaffold for safe transplantation, the desire to develop an ECM scaffold devoid of cellular content requires vigorous physical and chemical processes that may compromise the micro-topography, chemical content, and mechanical strength of the processed natural substrate that is essential to support cell growth.

Many approaches for tissue decellularization have been investigated, but there is still no consensus on the ideal method of bone decellularization technique that would produce an appropriate ECM for osseous regenerative medicine [12, 14]. The aim of this study is to investigate the efficacy of a novel decellularization technique for producing bovine cancellous bone scaffold and compare its physicochemical, mechanical, and biological characteristics with demineralized cancellous bone scaffold in an *in-vitro* study.

## Materials and methods

### Preparation of bovine scaffolds

#### Procurement of bovine bone

Cancellous bone blocks were harvested from fresh bovine femoral head obtained from one 24-month old calf from a local slaughterhouse. The whole femur was transported to the laboratory immediately and stored at -80°C until further use. Briefly, muscular soft tissue attachments around the femur were removed using a periosteal elevator. The femur head was separated from the shaft using a bone saw (JG210 Bone Cutting machine; Shandong China), and the long bone shaft was discarded. The outermost cortical layer of the bone surrounding the femoral head was removed, leaving behind the cancellous part. The cancellous part of the femoral head was then shaped in three bone block forms.

#### Processing of bovine cancellous bone scaffolds

The cancellous bone blocks were cleaned thoroughly in distilled water using a high-pressure water-jet spray with pressure not exceeding 160 psi. Delipidation was next performed by immersing the bone blocks in a mixture of alcohol-chloroform (1:1) for 24 h with a gentle shaking speed of 50 rpm at room temperature. The bovine bone blocks were then immersed in deionized water and rinsed thoroughly 3 times at 10 min intervals to remove the residual alcohol and chloroform. The bone blocks were then transferred to fresh distilled water and maintained in continuous rotation at 80 rpm (Shaker, SK-02, Zenith Lab co, China) for 24h to achieve complete removal of alcohol and debris trapped in the deep pores. Overnight washing in mild rotation produced clean bone samples without any visible red blood cell contamination or the presence of fat. The washed and defatted bone blocks were then randomly divided into two groups; the first group was processed to produce a lyophilized demineralized bovine cancellous bone (DMB) scaffold. The second group was treated to produce a lyophilized decellularized bovine cancellous bone (DCC) scaffold. A third group comprises non-processed native bovine cancellous bone that acts as a control.

In Group I, the defatted bovine bone blocks were subsequently demineralized by immersion in 0.6 M HCL for 24h with gentle rotation. At the end of acid treatment, these “demineralized bovine cancellous bone” (DMB) scaffolds were rinsed with sterile deionized water 3 times at 10 min intervals to ensure maximum removal of the residual acid. These steps were performed under continuous shaking at room temperature in a rotatory shaker.

In Group II, the defatted bovine bone blocks were decellularized, beginning with partial deproteinization by treating the bone in 4% sodium hypochlorite (NaOCL) for 24h with gentle shaking at room temperature. The bone blocks were then washed thoroughly with deionized water for 72h to eliminate residual chemicals. This was followed by rinsing in phosphate-buffered saline for 2h (PBS, Sigma Aldrich, USA) and followed by sequential washes in 0.01, 0.1, and 1% Sodium dodecyl sulfate (SDS) for a total period of another 72 h. The SDS-treated bone scaffolds were next exposed to 1% Triton X-100 (Sigma Aldrich, USA) in deionized water to remove the residual SDS and cellular debris. The treated scaffolds were then subjected to enzymatic treatment at the end of the Triton X-100 treatment procedure. They were immersed in a solution of DNase (0.2mg/ml; Sigma, USA) and RNase (1μg/ml; Sigma, USA) in PBS at 37°C with continuous shaking using a magnetic shaker (Labnet, USA). The enzyme mixture was replaced every 12h with freshly prepared DNase/RNase, and treatment was continued for 1 week. For residual nucleic acid quantification, samples were retrieved at specific time points; at days 1, 4 and 7 post exposure to DNase/RNase solution. Unprocessed native bone and decellularized bone samples without DNase/RNase exposure signify day 0 and both served as experimental controls. At the end of the enzymatic treatment, these “decellularized bovine cancellous bone” (DCC) scaffolds were rinsed thoroughly with sterile deionized water for 3h with intermittent changes of water.

#### Lyophilization and gamma sterilization

**Fig 1:**
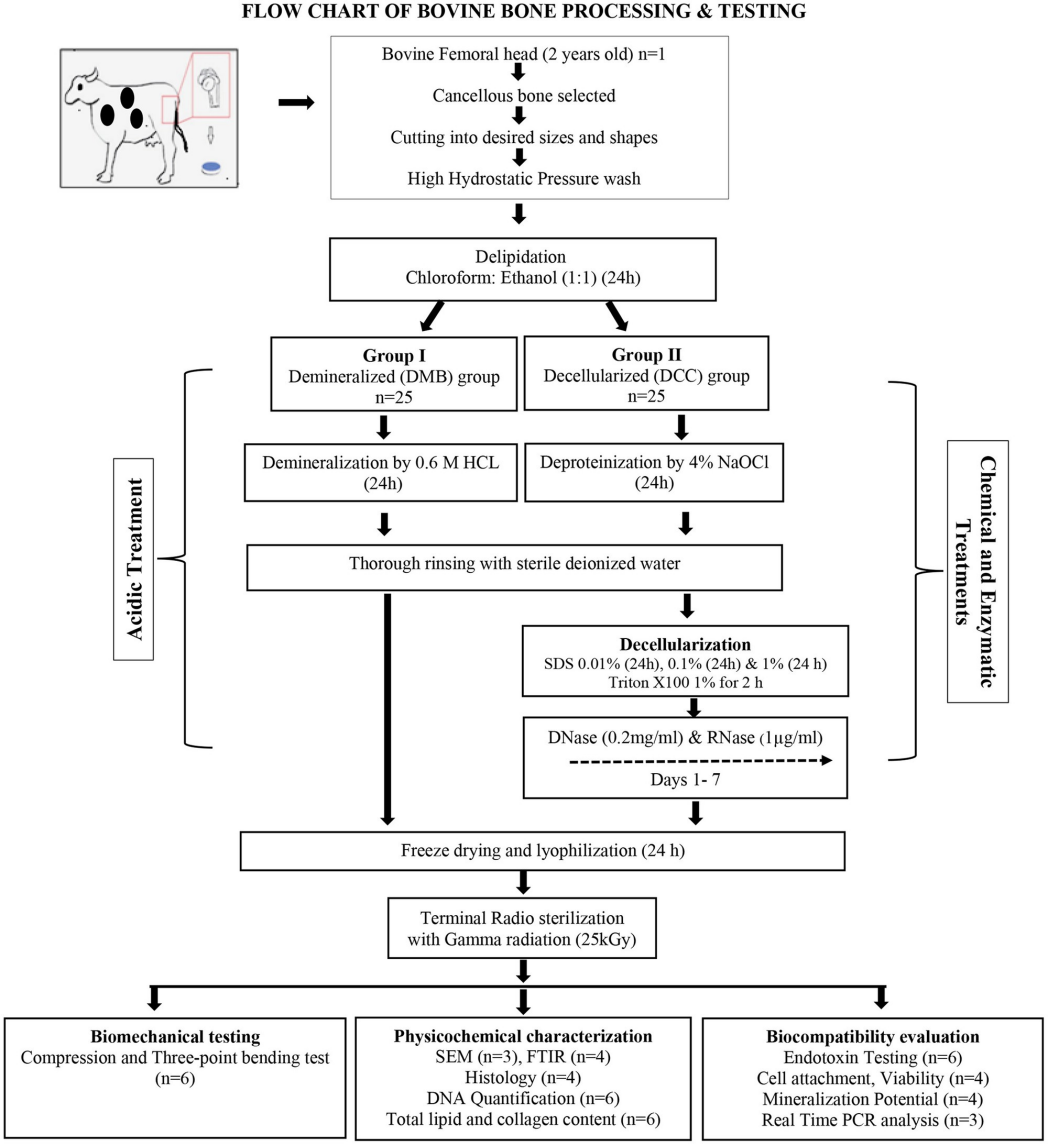
Bone processing steps. Schematic diagram depicting the sequential processing steps of producing lyophilized scaffolds of demineralized bovine cancellous bone (DMB) and decellularized bovine cancellous bone (DCC).

Both Group I DMB scaffolds and Group II DCC scaffolds were deep-freezed at -80°C for 4 h and lyophilized using Vir Tis BenchTop Pro with OmnitronicsTM (SP Scientific, PA, USA). The temperature of the lyophilizer was set at −40°C, and 7x10-2 milliBar pressure and the entire procedure lasted for 24h. Following lyophilization, the DMB scaffolds and DCC scaffolds were packed separately in double-layer plastic pouches and were radio-sterilized using gamma radiation at 25 kGy. The main sequential processing steps for producing DMB and DCC scaffolds in this study are shown in Fig 1.

To examine the differences in performance as a bone graft substitute between DMB and DCC scaffold, we evaluated their efficacy in terms of removal of all cellular materials, including nucleic acids and lipids, porosity, preservation of proteins and minerals, mechanical strength, as well as *in vitro* cytotoxicity, substrate support for osteoblast growth, osteogenic gene expression and mineralization potential of ECM.

### Physicochemical characterization of bovine scaffolds

#### Histological analysis

Samples from radio-sterilized DMB and DCC scaffolds and control native bovine cancellous bone blocks were fixed in 10% neutral buffered formalin (Thermo Fischer Scientific, USA), and decalcified using ready-made decalcification solution (Shandon^™^ TBD-1^™^ Decalcifier, Thermo Fischer Scientific, USA) with periodic examination for softening of the samples. Decalcification was performed in both DMB and DCC samples to prepare good-quality paraffin sections. The decalcification period for DMB samples, however, was shorter than for DCC samples. Upon completing the decalcification process, the bone samples were rinsed in running tap water thoroughly for 2 h followed by paraffin embedding and sectioning into 7μm thick sections. Bone sections were stained with nuclear dye Hematoxylin and the counterstain Eosin (Sigma-Aldrich). Images of tissue sections were captured using an inverted microscope (Olympus IX73, Japan).

#### Scanning electron microscopy with energy-dispersive X-ray spectroscopy (SEM-EDS)

Surface topography and surface elemental analysis was performed using SEM-EDS. The DMB and DCC scaffolds and the native controls shaped in 5 x 2 x 2 mm sizes were coated with gold using Quorum technologies SC7620 before SEM imaging using Tescan VEGA 3 XMU. Elements from three random regions across the scaffold samples were detected using Oxford Instruments X-max 50 EDS detector.

#### Evaluation of lipid content

The residual lipid content present in the lyophilized DMB and DCC scaffolds was estimated by Oil Red O quantification. About 100 mg of lyophilized gamma sterilized DMB and DCC scaffolds were incubated in 0.5 mL of Oil Red O solution (Sigma, USA) for 15 min at room temperature. Native control bone blocks served as control. The scaffolds and native bone blocks were washed in deionized water four times, followed by extraction of the oil red stain with 0.25mL 100% isopropanol, and the absorbance was measured at 490 nm using a multilabel plate reader (Multiskan TM GO, Thermo Scientific, USA).

#### Quantification of total collagen

The post-treatment collagen content in the DMB and DCC scaffolds was quantified using the Total Collagen Assay Kit (Abcam, USA), following the manufacturer’s instructions. The assay is based on alkaline hydrolysis of the bone samples releasing free hydroxyproline, which is later oxidized and quantified at OD 560 nm. All procedures were carried out in triplicates.

#### Fourier-transform infrared spectroscopy (FTIR)

The FTIR-ATR spectra of the DMB and DCC scaffolds were collected using Jasco FT/IR-6300 (Tokyo, Japan). Spectra were recorded with a resolution of 2 cm−1 with 20 scans over the range 4000–400 cm−1 at a constant temperature of 25°C. Three replicates of each sample were developed. Data analysis was performed using Origin Pro 8.5 software, and the presence of the ECM organic components, including collagen, fat, and carbonate, were evaluated. Spectra analysis was conducted in the attenuated total reflection (ATR) sampling mode and baseline corrected.

#### Quantification of residual nucleic acid in DMB and DCC scaffolds

Residual DNA/RNA content in lyophilized DCC scaffolds was determined using a DN/RN easy Blood and Tissue kit (Qiagen, USA). The lyophilized DCC scaffolds (n = 6) were pulverized, and 100mg of scaffold powder was used for DNA/RNA extraction. Total dsDNA/RNA was extracted following the manufacturer’s protocol with modifications at days 0, 1, 4, and 7 of DN/RNase treatment, respectively. The quality and concentration of the extracted total DNA and RNA were measured using the NanoDrop ND1000 (Thermo Scientific, Waltham, MA, USA) and expressed as nanograms per milligram of ECM dry weight. The extracted DNA was further loaded in 1% agarose gel run along a 100 bp DNA ladder to check the residual DNA fragment length. Residual total DNA content in lyophilized DMB scaffolds was determined using the above described method. Samples from native bovine bone blocks served as the control.

### Biomechanical characterization of bovine scaffolds

#### Compression test

Lyophilized DMB and DCC scaffolds were subjected to Quasi-static compressive tests in an air-dry condition. A universal testing machine, Model 5 ST (Tinius Olsen Single Column Testing Systems, USA), equipped with a 5kN load cell, was used for the analysis. Specimens were tested at a 1 mm/min crosshead speed. All specimens were loaded until compressive failure. Native control bone samples served as control.

#### Three-point bending test

Lyophilized DMB and DCC scaffolds shaped into 25mm x 5 mm x 2 mm sizes were prepared for a three-point bending test. A total of 6 bone samples in each group were tested using three-point bending by a universal testing machine, Model 5 ST (Tinius Olsen Single Column Testing Systems, USA), at a 1-mm/min crosshead speed. The failure point of each specimen was determined by analysis of the flexural stress-strain curves. Native control bone samples served as control.

### Biological evaluation of bovine scaffolds

#### Human osteoblast culture

Human osteoblast cells (HOB) used for biocompatibility evaluation were obtained from Addexbio (Cat no: P0004010; USA). The cells were cultured in a complete medium containing Dulbecco’s modified Eagle’s medium–Ham F12 (DMEM F12, Gibco^®^, USA) supplemented with 10% fetal bovine serum (FBS) and 1% antibiotics (Sigma, USA) in a humidified atmosphere at 37°C and 5% CO_2_. The medium was replaced every three days, and cells were passaged on approaching 80–90% confluence, using 0.25% trypsin-EDTA solution (Sigma, USA). HOB cells harvested in passages 3–6 were used for the experiments in the study.

#### Endotoxin testing

Before initiating the *in vitro* experiments, the DMB and DCC scaffolds were tested for endotoxin contamination using the chromogenic LAL (Limulus amebocyte lysate) assay (Lonza, Basel, Switzerland). The assay was performed according to the manufacturer’s instructions.

For the preparation of the samples, gamma-sterilized DMB and DCC scaffolds were incubated in endotoxin-free water (1 mL) for 24 h at 37°C to extract any potential endotoxin release from the bone scaffolds. After incubation, 50 μL of the supernatant was employed in the LAL test. A standard curve was prepared using the positive controls provided by the manufacturer within the LAL kit, and pure endotoxin-free water was used as the negative control.

#### *In vitro* cell viability

Direct and indirect methods were used to evaluate the biocompatibility of DMB and DCC scaffolds, and the amount of metabolically active cells was quantified by XTT assay (Roche Diagnostics, Mannheim, Germany). For the direct method, cells were seeded directly on the scaffolds at a density of 5x10^4^ cells/scaffold and incubated for a period of 24-48h. Before viability testing, the fixation rings were removed, and the scaffolds were transferred to new 12-well multiwell plates. The scaffolds were then incubated with XTT reagent for 4h at 37°C. 100μl replicates of the supernatant were then transferred into a 96-well plate, and absorbance was measured at 450 nm. For the indirect method, HOB cells at a density of 3 x 10^3^ cells were seeded on 96 well culture plates and incubated overnight for cell adherence. On the same day, scaffolds along with fixation rings were incubated with a complete medium overnight. The medium was aspirated out from the osteoblasts cells, replaced with the scaffold extracts (conditioned media), and incubated further for 48h. XTT assay was performed to assess the effect of scaffold extracts on cell proliferation and viability. In both methods, the culture medium with scaffolds holding the glass rings served as the negative control.

#### Cell seeding on scaffolds

DMB and DCC scaffolds were placed into 12-well multiwell plates (Sigma Aldrich, USA). The scaffolds were weighed down with sterile autoclaved stainless rings to keep them in position and prevent them from floating in the cell culture medium. Before seeding the cells, the scaffolds were incubated with a complete culture medium overnight in a CO_2_ incubator. The medium was then aspirated out, and the scaffolds were loaded slowly with cells in the center at a concentration of 5.0 × 10^4^ cells per scaffold in a total volume of 50-75ul. The multiwell plates were then incubated carefully without movement for initial cell attachment for 2-3h. The final culture volume was then made up to 1–2ml, and the cell-seeded scaffolds were further incubated for 2–3 days for cell attachment.

#### DAPI staining

The stainless steel rings were carefully removed from the cell-seeded DMB and DCC scaffolds, and the scaffolds were placed onto new culture wells. The scaffolds were then carefully washed three times with sterile PBS and stained with a mounting medium with DAPI (Abcam, USA) for 5–10 mins and observed under a fluorescence microscope (Olympus, Japan) for cell attachment in the first 24 hrs. Images were captured using fluorescence and confocal microscopes (Nikon Eclipse Ti-S, Nikon Instruments Inc., USA).

#### Scanning electron microscopy (SEM) of cell-seeded scaffolds

Osteoblast cell-seeded DMB and DCC scaffolds were subjected to SEM imaging. Briefly, cells were seeded on scaffolds and maintained in a complete DMEM-F12 medium for 7 days. The cell-seeded scaffolds were then fixed in 2.5% glutaraldehyde for 1 h, followed by progressive dehydration in ethanol and coated with gold using Quorum technologies SC7620 prior to SEM imaging using Tescan VEGA 3 XMU.

#### Mineralization study

HOB cells seeded onto DMB and DCC scaffolds were evaluated for their mineralization potential to assess their differentiation potential. Both DMB and DCC scaffolds were seeded with HOB cells at a density of 5 × 10^4^ cells/scaffold placed on 24-well plates, in the presence/absence of osteogenic factors (10^-8^M dexamethasone, 10mM beta glycerophosphate, and 50 μg/ml ascorbic acid). Matrix mineralization was evaluated at two time points, 7 and 14 days. Calcium deposition by the osteoblast cells was determined by Alizarin Red S staining (ARS). HOB cells seeded in the presence or absence of osteogenic factors served as a control in the experiment.

At the specified time points (days 7 and 14), the cells and cell-seeded scaffolds were washed in PBS and fixed in 4% paraformaldehyde solution for 15 min at room temperature. The cell/scaffold samples were stained with 40mM Alizarin Red S solution for 20 min at room temperature with gentle rocking. After removal of Alizarin Red S solution, the cell/scaffold samples were washed three times with deionized water, and the stained cells and scaffolds were imaged using an inverted phase-contrast tissue culture microscope (Olympus, CKX 41, NY, USA).

For calcium quantification, 10% acetic acid (v/v) was added to the Alizarin Red stained cells/scaffolds and incubated for 30 min at room temperature with agitation. Cells were scraped out from the culture wells, vortexed for 30 s, and incubated for 10 min at 85°C, whereas the cell-seeded scaffolds were directly vortexed and incubated at 85°C. Samples were centrifuged for 15 min at 12000 rpm, and the cell lysates were then collected as supernatant and quantified using a plate reader at absorbance 405 nm. A standard calibration curve for alizarin dye was prepared to quantify total calcium release.

#### Gene expression of osteogenic markers by real-time RT PCR

HOB cells grown on the DMB and DCC scaffolds for 14 days were evaluated for the expression of osteogenic genes that include alkaline phosphatase (ALP), osteocalcin (OC), and Runt-related transcription factor 2 (RUNX-2). The total RNA was extracted using the RNeasy kit (Invitrogen, USA) following the manufacturer’s instructions. The quality and concentration of the RNA samples were measured using the NanoDrop ND1000 (Thermo Fisher Scientific, USA). First-strand cDNA was synthesized by reverse transcriptase using the Highscript cDNA synthesis kit (Thermo Fisher Scientific, USA), and the expression of osteogenic markers was quantified using 5X FIREPOl SYBRGreen Mix (Solisbiodyne). The gene-specific primer sequences were used for the reaction, and glyceraldehyde-3-phosphate dehydrogenase (GAPDH) was used as an internal control for data normalization. HOB cells grown in the absence of an osteogenic medium served as control. The qPCR amplification was conducted as follows: initial denaturation at 95°C for 10 min, followed by 40 cycles at 95°C for 30 s, 60°C for 1 min, and 72°C for 1 min. The reaction was performed using StepOne Thermocycler (Applied Biosystems, USA), and the results were quantified using the ΔΔCt relative quantification method.

### Data and statistical analysis

Statistical analysis was carried out using GraphPad Prism 5. Data were expressed as mean ± SEM. One-way analysis of variance (ANOVA) with Bonferroni’s post hoc test was applied to identify the differences between the study groups, and an unpaired t-test was used whenever differences between two groups were studied. A p-value of p < 0.05 was considered to be statistically significant.

## Results

### Gross morphology of processed bone scaffolds

**Fig 2:**
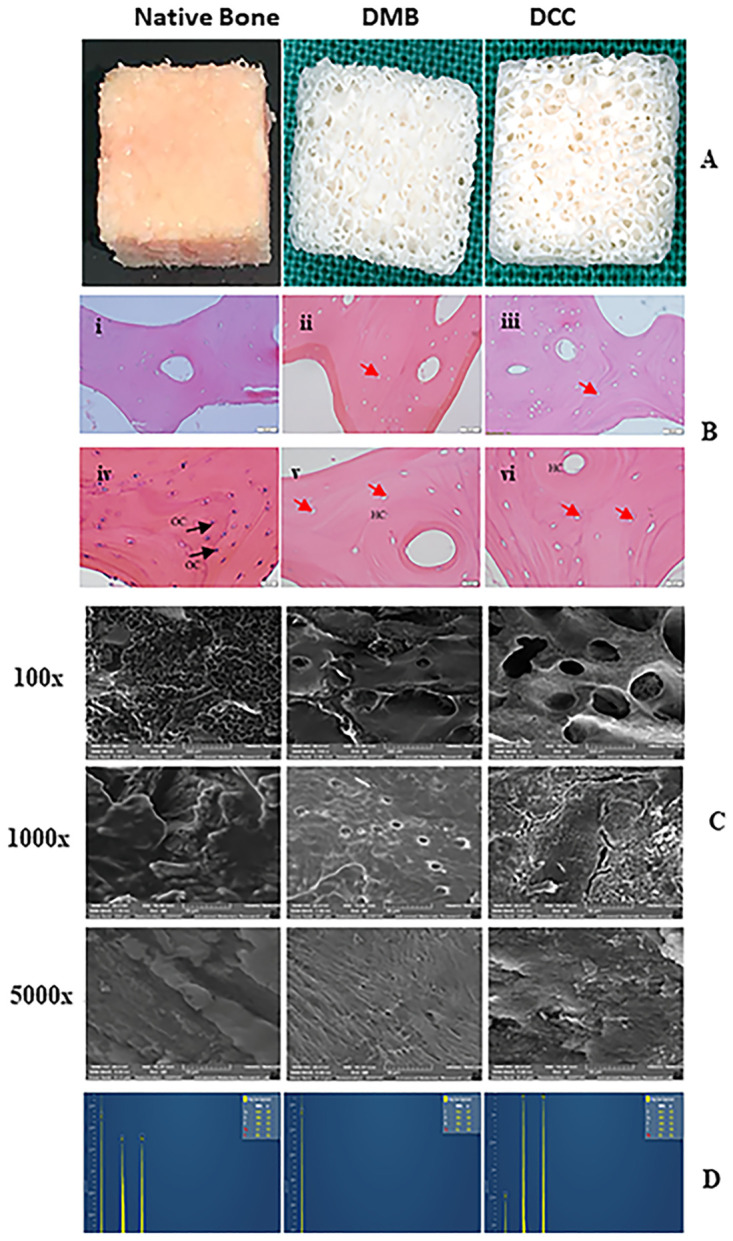
(A). Gross appearance of DMB and DCC scaffolds. The DMB and DCC scaffolds appeared as a clean white sponge, with a gross porous structure compared to untreated native control bone. (B). Hematoxylin and Eosin staining images of bone scaffolds. (i, iv) Control native bone; (ii, v) DMB scaffold; and (iii, vi) DCC scaffold. Prior to sectioning, the DMB and DCC samples were both decalcified using the Shannon TBD-1 Decalcifier. Staining with Hematoxylin and Eosin showed intact osteocytes nuclei in control native bone (black arrows) while nuclei were largely removed from the DMB scaffold cells and absent in the DCC scaffold, resulting in empty lacunae with no visibly-stained nucleus within the lacunae (red arrow). HC- Haversian canal, OC-osteocyte. The scale bar is 50 μm (upper panel) and 20 μm (bottom panel). (C). Representative SEM images of DMB and DCC scaffolds compared to native control. Magnification at 100x, 1000x and 5000x is indicated. (D). EDS surface analysis of the scaffolds. The EDS surface analysis technique used a representative spectrographic analysis showing the surface chemical elements detected in DMB and DCC scaffolds compared to native control.

The gross morphology of the processed DMB and DCC scaffolds was analyzed to examine the physical, chemical, and enzymatic treatment impact. Both scaffolds appeared white and clean, without any soft tissue attachments or debris within the pores. Effective removal of fat and red bone marrow was observed in both DMB and DCC scaffolds exhibiting a well-porous sponge-like morphology (Fig 2A).

### Histological analysis

Histological examination of lyophilized DMB scaffolds showed an intact general structure of cancellous bone trabeculae with preserved lacunae and some containing osteocytes. At the same time, the lyophilized DCC scaffolds also presented with an intact general cancellous structure with well-preserved empty lacunae, demonstrating numerous amounts of missing osteocytes. Both scaffolds exhibited contrasting histological features compared to the intact native bone matrix that demonstrated numerous amounts of osteocytes with well-preserved cell morphology (Fig 2Bi & 2Biv) in the lacunae. The naturally porous structure of the extracellular bone matrix was well maintained in both the DMB and DCC scaffolds. While the cellular morphology of the DMB bone showed deterioration of cellular components with the visible appearance of remnant pyknotic nuclei (Fig 2Bii & 2Bv), the number of empty lacunae without traces of nuclei were comparatively more numerous in DCC scaffolds (Fig 2Biii & 2Bvi).

### SEM-EDS evaluation of scaffold

Morphological assessment at the ultrastructural level of DMB and DCC scaffolds using SEM revealed the clean surface and pore architecture (Fig 2C) exhibiting explicit natural porous microstructure and three-dimensional interconnectivity compared to the native control bone samples. High magnification at 1000x and 5000x revealed the clean and bare surface morphology in both treatment groups compared to native bones, where the deeper bone structures were not visible and covered completely by layers of fat and debris. The protein phase (collagen fibril) was observed in the DMB scaffold, while clusters of calcified filaments (mineral phase) were visible in the DCC scaffold SEM micrograph. The untreated native bone (mineral and protein phases together) showed a compact surface morphology.

EDS surface analysis element composition detected sodium, calcium, phosphorus, and oxygen in the DMB and DCC scaffolds (Fig 2D). The quantitative chemical analysis obtained is shown in Table 1 (n = 3). There was a significant reduction in the amount of calcium in both DMB (0.1 ± 0.0; p < 0.001) and DCC (29.0 ± 0.3; p < 0.001) scaffolds when compared to the native control bone samples (41.8 ± 0.8). However, the percentage of calcium in the DMB group was significantly lower than that of the DCC group (0.1 ± 0.0 vs. 29.0 ± 0.3; p < 0.001; Table 1). The amount of phosphorous in DCC scaffolds (14.8 ± 0.2) was closer to native bone (16.6 ± 0.5), while it was undetectable in DMB scaffolds.

**Table 1:** Percentage of surface chemical elements detected in DMB and DCC scaffolds compared to native control.

	Element composition (Wt %)			
Scaffold type	Ca	P	Na	O
Native Control	41.8 ± 0.8	16.6 ± 0.5	1.2 ± 0.3	39.8 ± 1.0
DMB	0.1 ± 0.0 ***	-	-	28.3 ± 0.4
DCC	29.0 ± 0.3***	14.8 ± 0.2	0.5 ± 0.1	41.5 ± 0.5

DMB—demineralized bovine cancellous bone; DCC-decellularized bovine cancellous bone (** p< 0.001)

**Fig 3:**
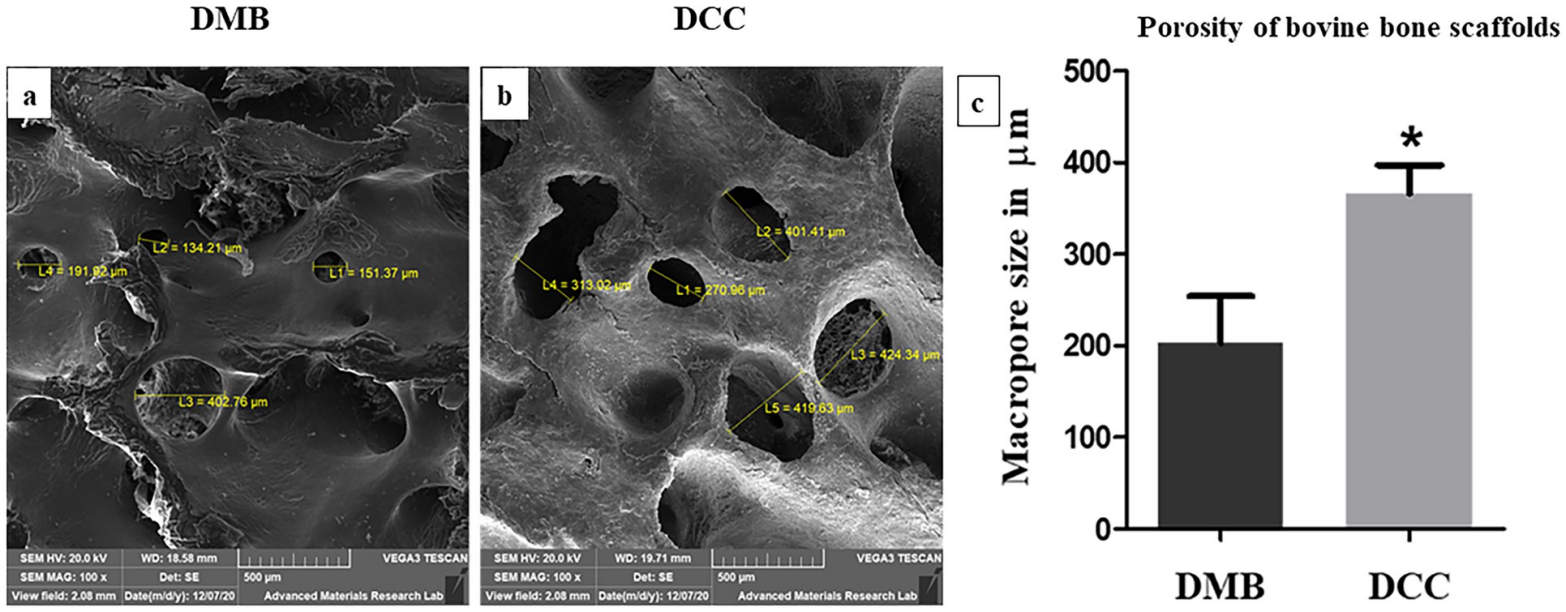
Variations in pore morphology related to scaffold treatment. Representative images showed pore sizes on the surface of the DMB (a) and DCC (b) scaffold. DCC scaffolds present significantly wider pore diameters with richer interconnectivity compared to DMB at 100x magnification (c) (*p < 0.05; n = 4).

When the porosity between DMB and DCC scaffolds was compared, the DCC scaffolds generally showed higher porosity (Fig 3a & 3b). Under 100x magnification, DCC scaffolds showed a relatively larger pore size (365.87 ± 62.2 μm; p < 0.05; Fig 3c), showing deeper porosity with obvious interconnectivity compared with DMB scaffold micro-architecture (202.92 ± 102.11 μm). The porosity depth in the DMB scaffold was generally not very clearly seen. No feature of microfractures was observed at 5000X magnification on the surface and deeper pores of both DMB and DCC scaffolds.

### Lipid content

**Fig 4:**
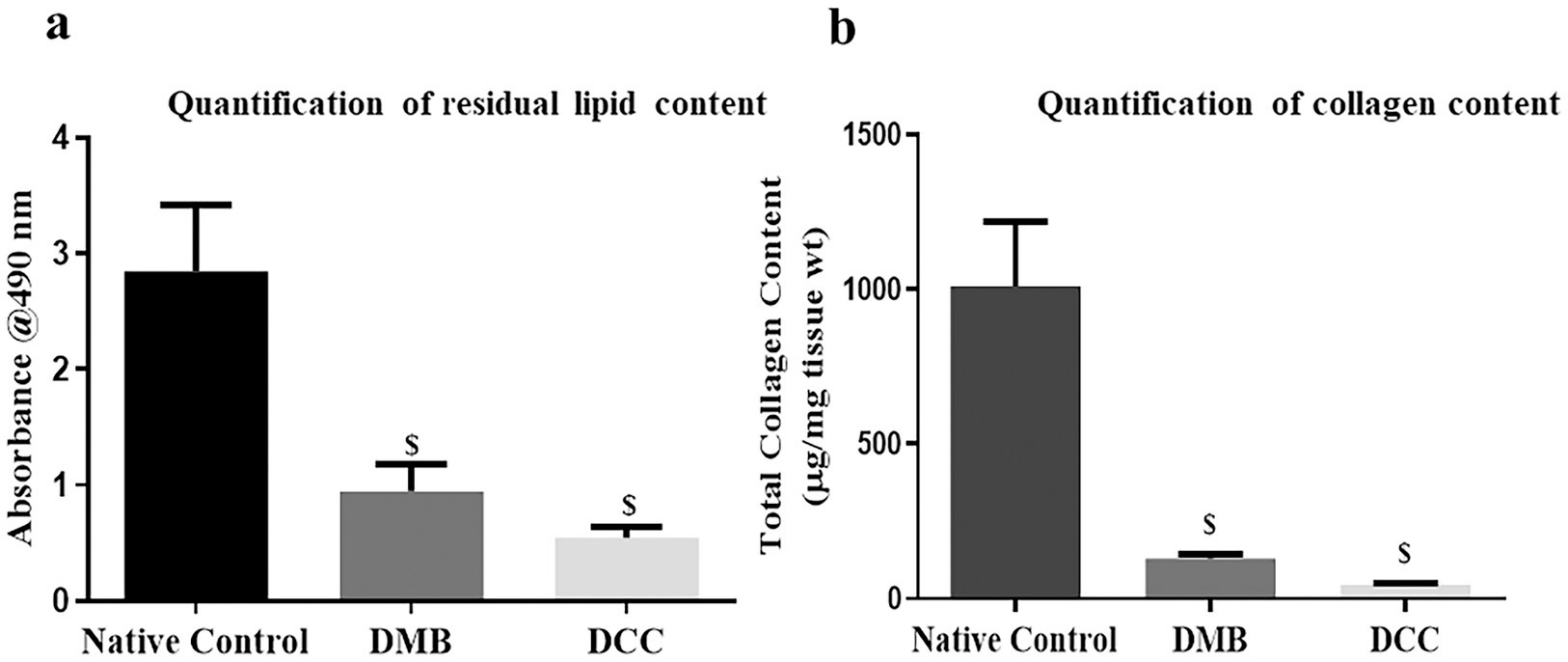
(a). Lipid quantification in scaffolds. Quantification of residual lipids by Oil Red O staining in DMB and DCC scaffolds compared to native control. Values are expressed as mean ± SEM (n = 6). Statistically significant difference of ^$^p < 0.05. (b). Quantification of total collagen content in DMB and DCC scaffolds. A significant reduction was observed in total collagen content in both DMB and DCC scaffolds compared to native control. Values are expressed as mean ± SEM (n = 6; ^$^p < 0.05).

Oil Red O staining confirmed the efficient removal of residual lipid content in DMB and DCC scaffolds. Oil Red O stain combines with triacylglycerols to form jacinth lipid droplets which can be quantified spectrophotometrically. Compared to native control bone, DMB and DCC scaffolds showed significant reduction in total lipid content (p<0.05; Fig 4a).

### Collagen content

Total collagen assay revealed a significant reduction in the total collagen content of both DMB (131.22 ± 10.79 μg/mg ECM wt; p < 0.05) and DCC (45.45 ± 4.69 μg/mg ECM wt; p < 0.05; Fig 4b) scaffolds as compared to the native control bone (1010.73 ± 170.42 μg/mg ECM wt). However, there was no significant difference in the amount of total collagen removed when demineralization and decellularization treatments were compared.

### FTIR analysis of bone samples

**Fig 5:**
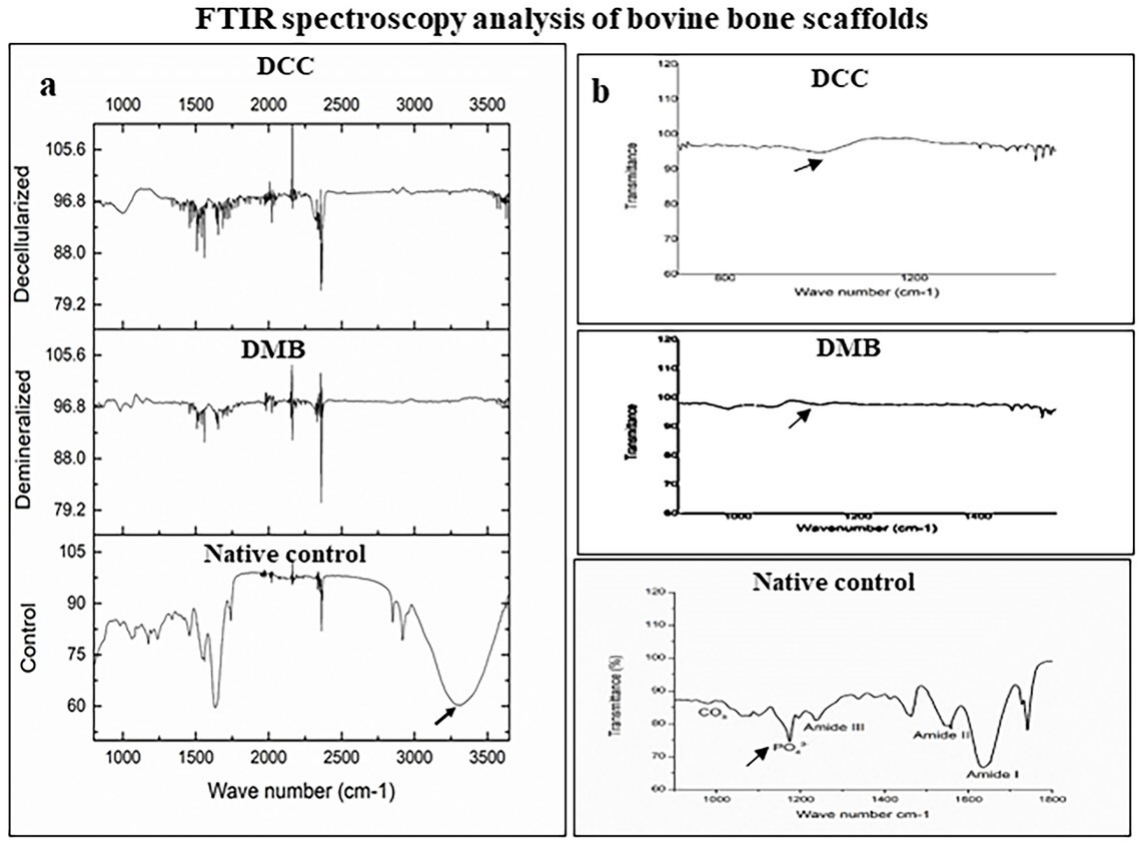
FTIR spectra of DMB and DCC scaffolds. A broad absorption peak of cholesterol is evident at 3412 cm −1 in native control bone, whereas a similar peak is absent in DMB and DCC scaffolds (a). FTIR Spectrum of DMB and DCC bone scaffolds compared to native control bone. Regions of interest are indicated: carbonate (850–890 cm-1), phosphate (900–1200 cm-1), amide I (1585–1720 cm-1), amide II (1500–1600–1), and amide III (1250–1350 cm-1) absorption peaks (b); n = 4.

The FTIR spectra recorded in the DMB and DCC scaffolds showed significant changes compared to the native control bone, and specific peaks of interest have been marked in the spectrum (Fig 5). A broad absorption peak of cholesterol is evident at 3412 cm −1 in native control bone but was not found in both DMB and DCC bone scaffolds suggesting the effective removal of fat from the bone samples by both treatment processes (black arrow; Fig 5a). Other regions of interest include collagen that is depicted as amide I (1585–1720 cm-1), amide II (1500–1600–1), amide III (1250–1350 cm-1); and carbonate (850–890 cm-1) and phosphate (900–1200 cm-1) are also demonstrated (Fig 5b). A credible decline in phosphate peak (black arrow) was observed post demineralization and decellularization treatments. Amide I results from peptide bond C = O stretch, amide II from mixed C–N stretch and N–H in-plane bend, and amide III results from mixed C–N stretch and N–H in-plane bend.

### Quantification of residual nucleic acid content in DCC bone samples

**Fig 6:**
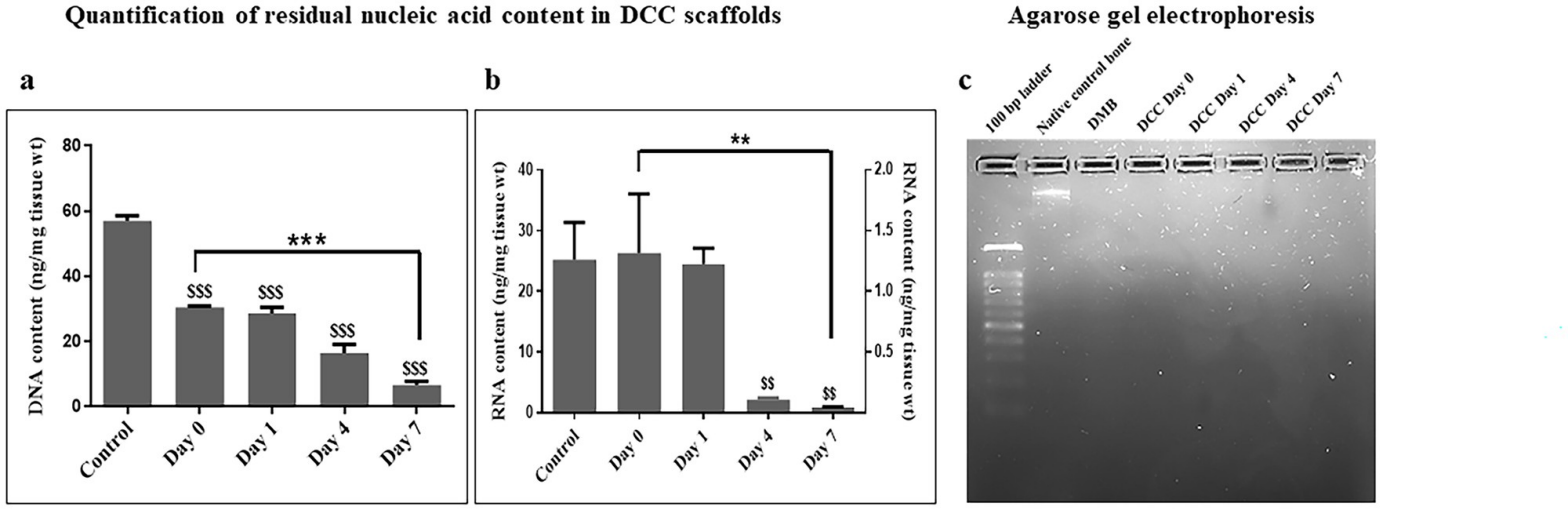
Quantification of the total nucleic acids in DCC scaffolds. The amount of residual DNA (a) and RNA (b) in the DCC scaffolds compared to native control bone and processed bone samples without DNase/RNase exposure; signifies day 0 is shown. Total residual DNA and RNA obtained were normalized by the dry weight of each bone sample and interpreted. Values are expressed as mean ± SEM (n = 6). Statistically significant differences of ***p < 0.001,**p < 0.01, ^$$$^p <0.05 and ^$$^p<0.0001 are indicated. (c) Agarose gel electrophoresis image of the total DNA extracted from DMB scaffold, DCC without exposure to DNase/RNase (day 0) and on days 1, 4, and 7 post-DNase treatment of DCC scaffolds compared to native control bone.

The quantification of nucleic acids in both native cancellous bone blocks and DCC scaffold is shown in Fig 6a and 6b. Total residual DNA/RNA obtained was normalized by the dry weight of each bone sample and interpreted. The average total DNA content obtained was 56.9 ± 2.37 ng/mg dry wt of ECM for native control bone and 30.4 ± 0.49 ng/mg dry wt of ECM (p<0.001) in DCC scaffold at day 0. The DNA content in the DCC scaffold was significantly reduced to 28.5 ± 2.69 ng/mg dry wt of ECM (p<0.001) on day 1, and further declined to 16.33 ± 3.68 ng/mg dry ECM wt (p<0.001) on day 4 and reduced to a low amount of 6.53 ± 1.57 (p<0.001) on day 7 of DNase treatment (Table 2).

**Table 2:** Nucleic acid quantification. Quantification of residual DNA/RNA content in DCC scaffolds after one week of DNase/RNase treatment compared to native control bone. The total DNA/RNA content of the processed demineralized and decellularized bovine bone samples on different treatment days (days 1, 4 and 7) is shown in comparison to day 0 of the treatment period with statistical significance.

	DNA Concentration (ng/mg dry wt of ECM ± SEM)	RNA Concentration (ng/mg dry wt of ECM ± SEM)
Native Control	56.9 ± 2.37	27.87 ± 11.05
Day 0	30.4 ± 0.49 ***	28.04 ± 15.56
Day 1	28.5 ± 2.69 ***	24.30 ± 4.70
Day 4	16.3 ± 3.68	0.10 ± 0.03 **
Day 7	6.53 ± 1.57 ***	0.05 ± 0.00 **

** p<0.01

*** p< 0.001

The average total RNA content was 27.87 ± 11.05 ng/mg dry wt of ECM for native control bone and 28.04 ± 15.56 ng/mg dry wt of ECM for the DCC scaffold. The RNA content in the DCC scaffold was reduced to 24.3±4.7 ng/mg dry wt of ECM on day 1, and further declined significantly to 0.1 ±0.03 ng/mg dry wt of ECM on day 4 (p<0.01) and finally reduced to a meager amount of 0.05 ± 0.03 ng/mg dry wt of ECM (p< 0.01) on day 7 of RNase treatment. There was a significant reduction in total RNA content from Day 0 to Day 7 of RNase treatment (Table 2; p<0.01). Agarose gel electrophoresis image revealed the presence of a visible DNA band of high molecular weight in the native control bone sample, whereas DMB and DCC scaffolds did not show the presence of any DNA bands (Fig 6c).

### Biomechanical analysis

#### Compression test

**Fig 7:**
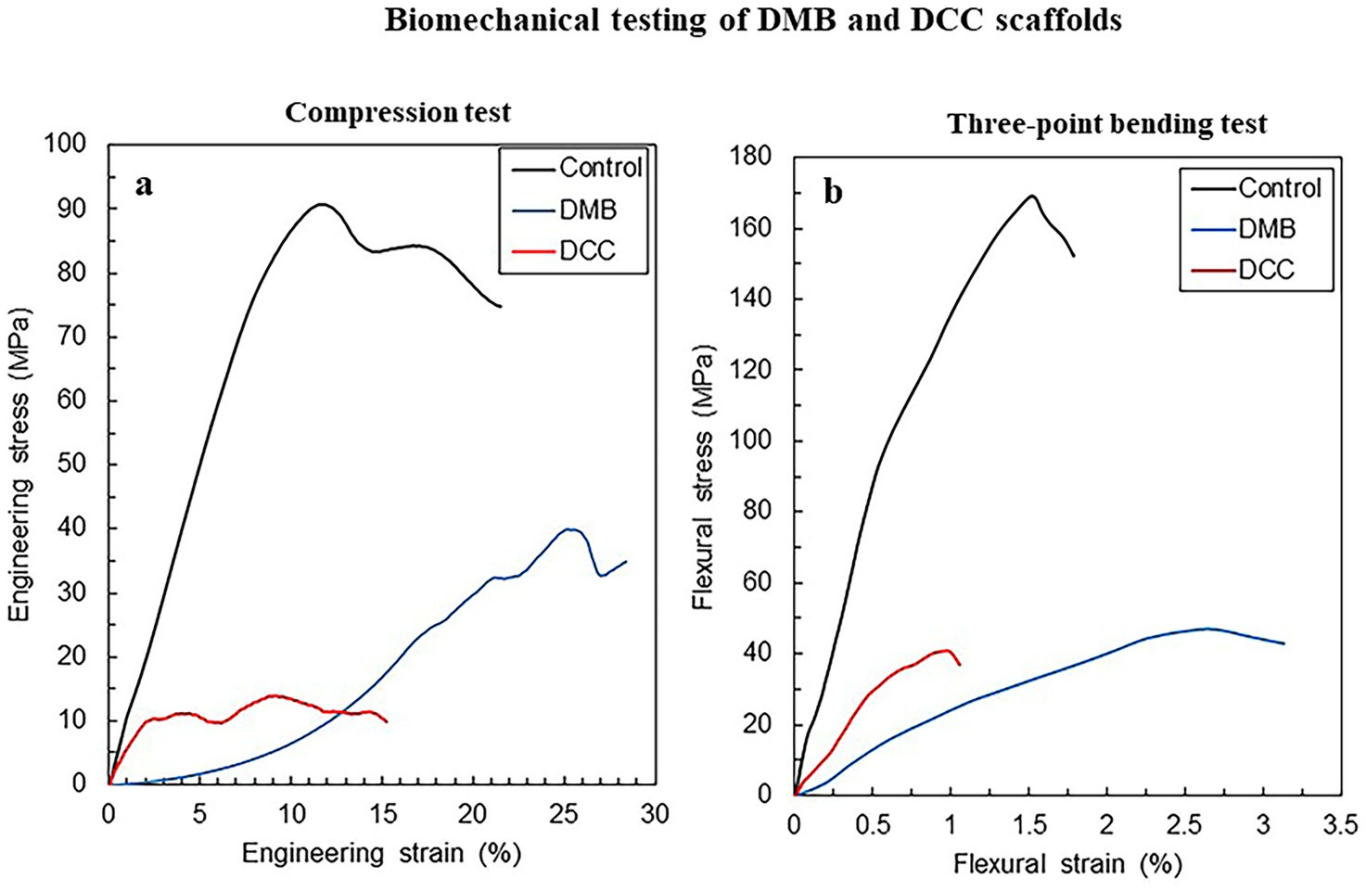
Stress-strain curve of scaffolds in the compression test. Representative images of compressive engineering stress and engineering strain (SS) curves of native control bone, DMB, and DCC scaffolds; n = 6 (a). Flexural-strain curve of scaffolds in three-point bending test. Representative images of flexural stress and strain curves of native control bone, DMB, and DCC scaffold; n = 6 (b).

Compressive engineering stress and engineering strain (SS) curves of all sample groups are shown in Fig 7. All samples exhibited a typical SS behavior of bone [15]: elastic and plastic regions. In the elastic region, the stress increases linearly with increasing strain, then a point in the linear relationship finally ends when the bone begins to yield. The yield point marks the beginning of plastic deformation, and then the plastic deformation occurs upon compressive test afterward. Since there is no significant boundary between elastic and plastic regions, 0.2% strain offset [16], i.e., 0.2% proof stress, was applied as the yield point in this study. The 0.2% proof stresses and the ultimate compressive stresses of control samples were higher than other scaffolds, while the DCC showed the lowest 0.2% and ultimate compressive stresses.

#### Three-point bending test

**Fig 8:**
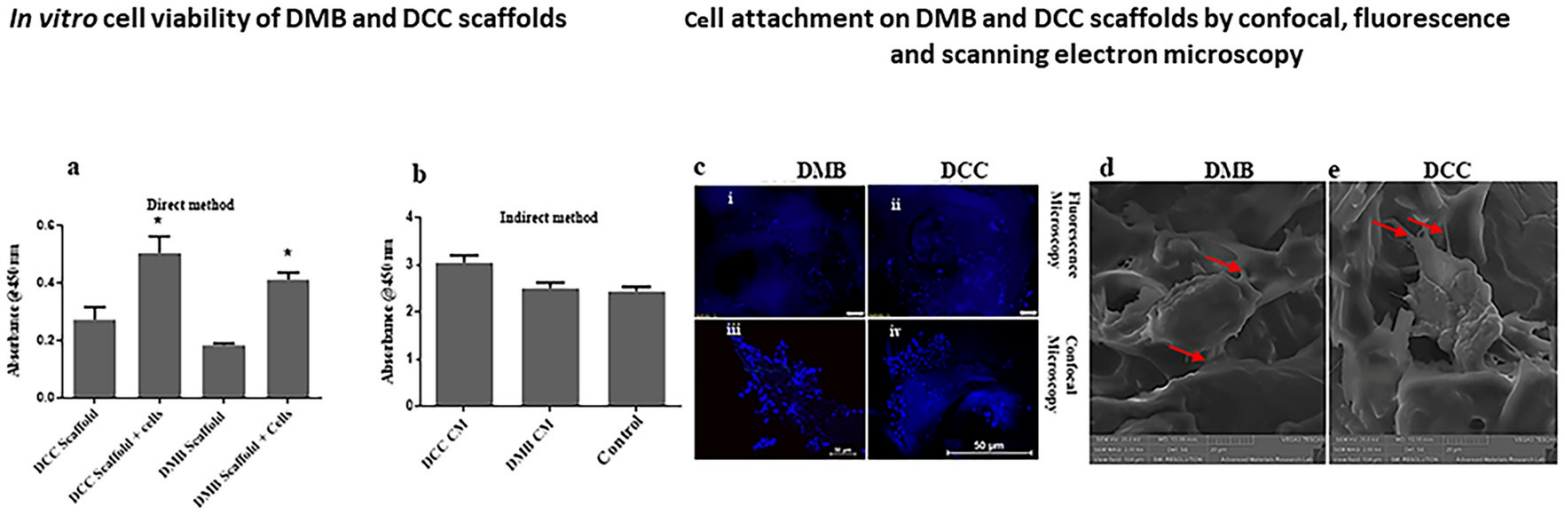
Cell viability studies employing DMB and DCC scaffolds. Bar charts indicate comparison between DCC scaffold vs DCC scaffold + cells and DMB scaffolds vs DMB scaffold + cells by direct method (a). Bar charts indicate comparison between DMB conditioned media (CM) and DCC conditioned media (CM) vs complete culture media (Control) by indirect method (b). *p<0.05 is indicated. n = 4; CM: conditioned media. (c). HOB cells seeded on DMB and DCC scaffolds showing optimum cell attachment by DAPI staining. (i & iii) DMB scaffolds and (ii & iv) DCC scaffolds; images were captured by fluorescence and confocal microscopy, respectively. Scanning electron micrograph of HOB cells seeded onto DMB (d) and DCC (e) scaffolds. The osteoblast shows a healthy morphology residing on the ECM with evidence of filopodia spread (red arrows).

The relation between flexural stress and strain of all the sample groups is shown in Fig 8. The flexural SS curves also exhibit elastic and plastic deformation behavior of bones and are similar to the SS curve of the compressive test. The maximum flexural stress of the DCC scaffolds was higher than the DMB, while the DMB had a higher total strain than the DCC. The average flexural elasticities of the native control, DMB, and DCC scaffolds are 17.2 GPa, 1.7 GPa, and 5.9 GPa, respectively.

#### Endotoxin testing

The endotoxin level present in both DMB and DCC scaffolds was observed to be 0.010025 and 0.015058 EU/ml, respectively, which is much below the FDA limit of 0.05 endotoxin units/ml [17].

#### *In vitro* cell viability

Cell viability study revealed that both DMB and DCC scaffolds supported the growth and proliferation of osteoblast cells. The present study addressed both the direct and indirect methods for evaluating the biocompatibility of the scaffolds. In the direct method, cell proliferation was significantly high in both DMB and DCC scaffolds compared to the respective unseeded scaffold controls (Fig 8a; p< 0.05). Although cell proliferation was observed to be higher in DCC scaffolds in comparison to cell-seeded DMB scaffolds, the difference was not significant, suggesting an equal efficacy for both scaffolds in supporting osteoblast proliferation. In the indirect method, cell proliferation in the presence of DMB and DCC scaffold extracts (conditioned media) were comparable with the complete culture (control) medium, suggesting no inhibition in growth and proliferation of cells upon exposure to either scaffold extracts (Fig 8b). The data suggest that the porous nature of the scaffolds provided ambient conditions for adhesion, colonization, and proliferation of osteoblast cells and enabled adequate nutrient supply.

#### Cell seeding and DAPI staining

DAPI staining of the cell-seeded scaffolds revealed that both DMB and DCC scaffolds are conducive substrates for cell attachment and spread. Osteoblast cell attachments were observed to be sufficient densities in DMB and DCC scaffolds (Fig 8c).

#### SEM of the cell-seeded scaffolds

SEM images showed good cell attachment capability of osteoblasts and their interaction with the extracellular matrix in the DMB and DCC scaffolds. After seven days of culture, osteoblasts seeded on DMB scaffolds showed a healthy appearance with filopodia extending to the ECM (Fig 8d). Similar cytoplasmic processes and osteoblast-substrate engagement were also visible in DCC scaffolds (Fig 8e). The presence of intact collagen fibrils within the porous microstructure offers cell attachment sites that favor the establishment of cell-matrix and cell-cell interactions through their extended filopodia.

#### Mineralization study

**Fig 9:**
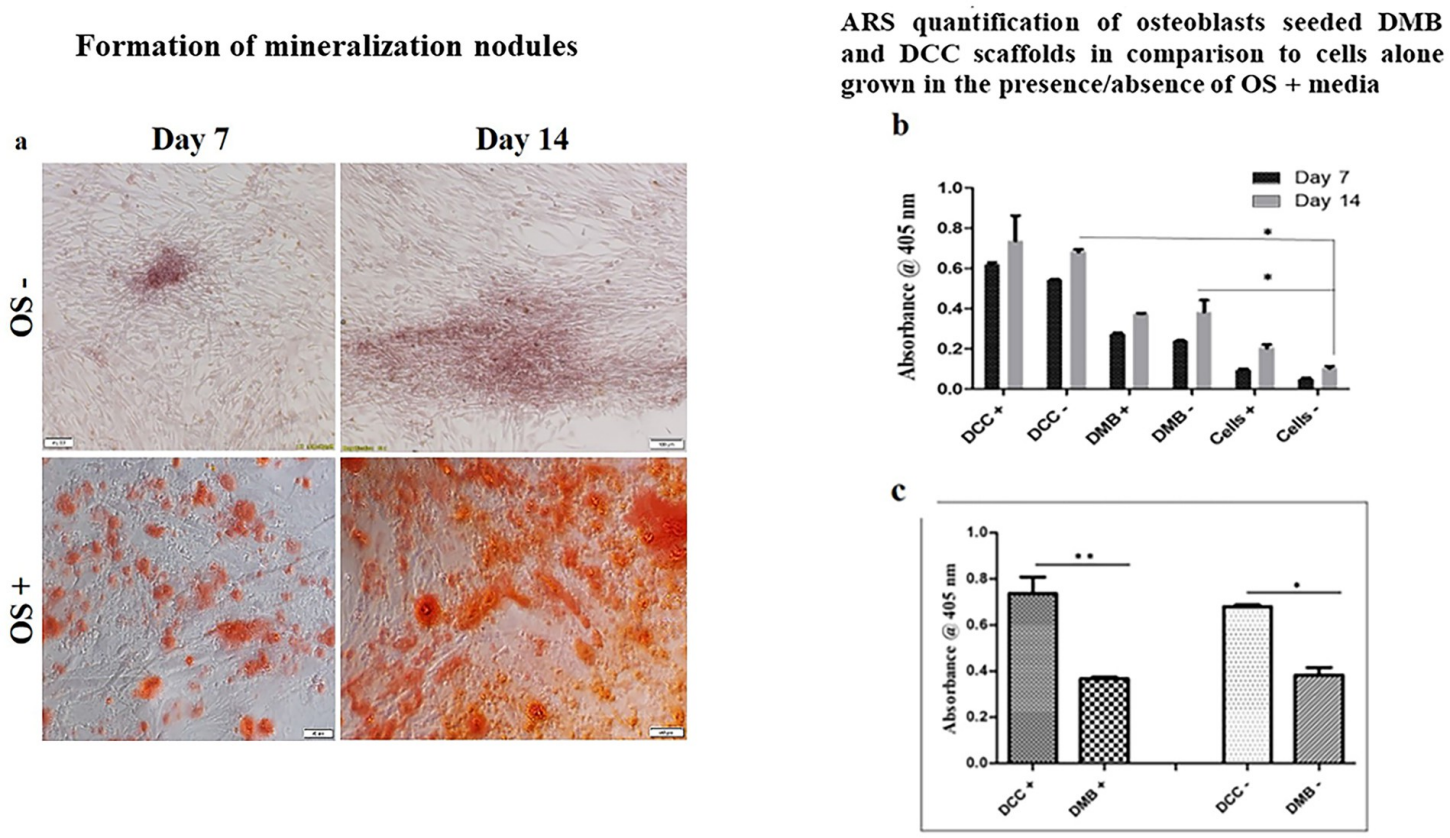
Formation of mineralization nodules. Photomicrographs of HOB cells stained with Alizarin Red S. HOB cells showed increased mineralized nodules in osteogenic medium (OS^+^) compared to cells without supplements (OS^-^). More significant mineralization was seen by day 14 (a). Quantification of alizarin Red S stained mineralized nodules in human osteoblast cells seeded on DMB and DCC scaffolds grown in the presence or absence of osteogenic induction media at days 7 and 14 in comparison to cells alone is shown; p<0.001 is indicated). The absorbance of the extracted Alizarin dye was measured at 405 nm. Data presented as mean ± SEM (n = 6) (b). Quantification of Alizarin Red S staining in osteoblast cell-seeded DMB and DCC scaffolds on day 14 in the presence and absence of osteogenic supplements. Data presented as mean ± SEM (n = 4) (c); p<0.001 is indicated.

The effect of scaffolds on inducing osteoblast mineralization was studied in the presence and absence of osteogenic media at two specific time points on days 7 and 14. Cells alone grown in the presence and absence of osteogenic media served as a control in the experiment. Alizarin Red S staining revealed nodule formation in cells supplemented with osteogenic media compared to those without any supplements. Also, more intense staining was observed on day 14 of cell culture than on day 7 (Fig 9a).

When the osteoblast cells seeded DMB and DCC scaffolds were studied for their mineralization potential, it was observed that DCC scaffolds favored enhanced differentiation of osteoblasts compared to DMB scaffolds in the absence of osteogenic supplements. Quantification of Alizarin Red stain on day 14 revealed that mineralization was more greater in cells seeded on DMB (p<0.001) and DCC scaffolds (p<0.001) even in the absence of osteogenic supplements (Fig 9b). When both the scaffolds were compared, mineralization was more abundant in cells seeded on DCC scaffolds than on DMB scaffold (p<0.001; Fig 9c) irrespective of the presence or absence of osteogenic supplements.

#### Gene expression of osteogenic markers

**Fig 10:**
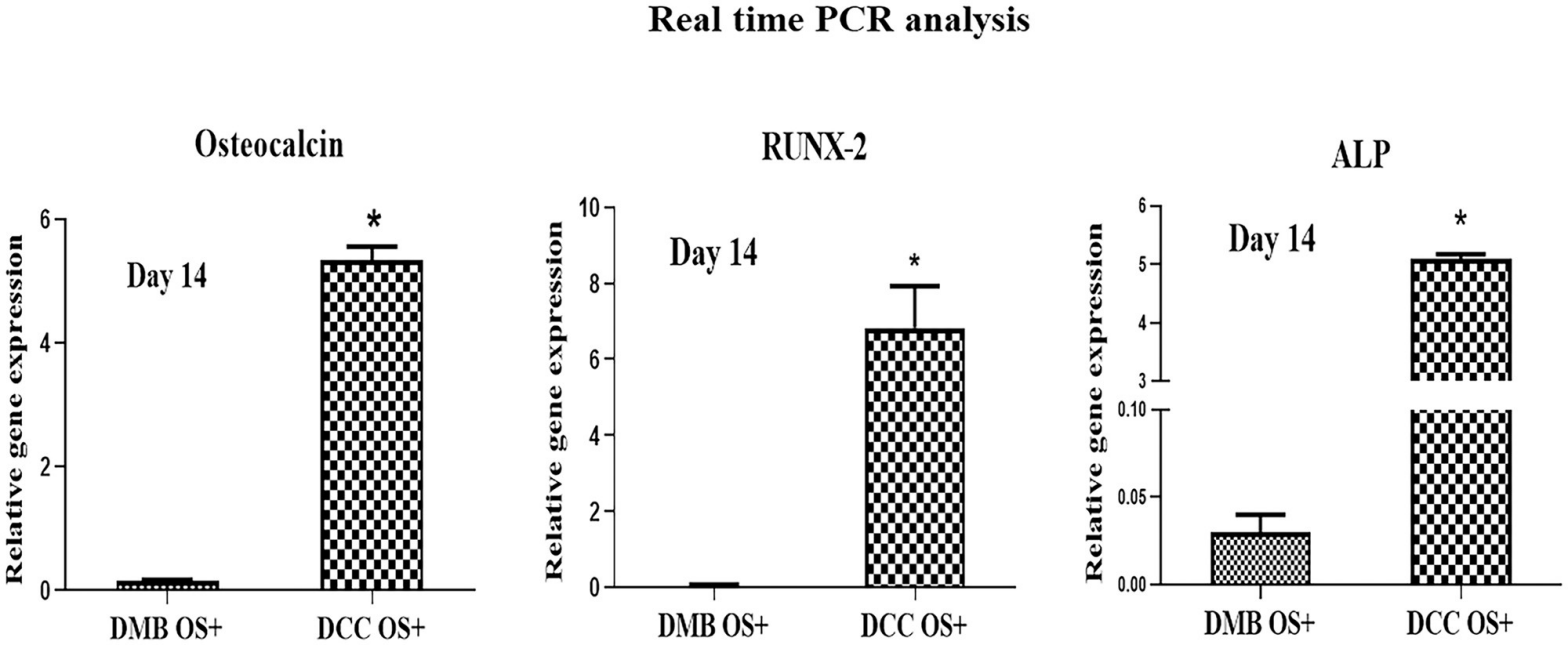
Gene expression during mineralization of human osteoblast cells grown on DMB and DCC scaffolds in osteogenic medium on day 14. The expression of genes was analyzed by real-time PCR. GAPDH expression was used as an internal control (n = 3).

Expression of the osteogenic markers such as alkaline phosphatase (ALP), osteocalcin (OCN), and RUNX2 in osteoblasts cultured on the DMB and DCC scaffolds for 14 days were analyzed by quantitative real-time PCR, in support of the findings from Alizarin Red S staining experiments. Expression of the osteogenic markers was significantly higher in cells grown on DCC scaffolds than in cells grown on DMB scaffolds for all three osteogenic markers (p<0.001; Fig 10). It was also observed that the expression for the early osteogenic marker, RUNX2, was higher compared to ALP and OCN markers.

## Discussion

While the use of demineralized bone matrix is common practice in dentistry, the application of decellularized bone substitutes is still lacking. However, current immunological issues related to the transfer of xenogeneic donor bone cells into the recipient are a rising challenge, and a search for more promising new methods to prepare natural matrices for safe tissue regeneration is needed [13]. In this study, the bovine femoral head was chosen as the source of starting material due to its spongy cancellous structure and composition suitable for dental applications. Indeed, the presence of marrow spaces within the structure of cancellous bone scaffold, and its large surface area, makes it very attractive to graft sites requiring less mechanical strength and as space filler is mostly preferred for repairing dental defects.

The unlimited supply of xenogeneic tissues has been the main driver in developing bovine bone scaffolds [18] While the innate immune responses following transplantation of processed animal tissues are known, the long-term adaptive T-cell response following the introduction of residual bovine genetic material into the human host has not been adequately addressed. This phenomenon may have often resulted in chronic inflammatory reactions at bovine bone graft implantation sites commonly encountered in clinical practice [13]. Thus, immuno-compatibility has become one of the essential properties in xenogeneic bone scaffold development. Clinical experience showed that the genotoxicity complications following the transfer of genetic material from donor tissue graft into recipient might manifest much later, often undetected within the context of biocompatibility, and the evidence needs the test of time. The only way to assure freedom from genetic transfer risk in the host is to eliminate all the donor cells present in the processed bovine bone graft in a technique called decellularization.

Extracellular matrix-based biomaterials created by decellularizing organs, bone tissues, cartilages or cell cultures are gaining popularity and there are many FDA-approved extracellular matrix-based materials that are generated through decellularization processes [18–20]. This study demonstrated a comparison between DMB and DCC with regards to mechanical and biological efficacy *in vitro*. The initial common preparation process for both scaffolds began with a high hydrostatic pressure wash which enabled the destruction of cell membranes and debris removal to facilitate subsequent alcohol-chloroform treatment in the deeper parts of the bone for defatting. For decellularization, the scaffolds were subjected to NaOCL bleaching agent, that can form complexes with proteins through hydrophobic interactions and solubilize cell membranes and membrane-bound proteins [21].

Although the literature does not encourage the use of detergents in bone cleansing processes, it is the concentration of the chemicals used, the duration employed, the ideal ambient environment temperature, and the speed of chemical stirring that determine the scaffold end products. Although certain reports draw our attention to the drawbacks of using acid/bases, SDS, DNases and hydrostatic pressure in bone tissue decellularization [19], the duration and concentration of chemicals used in the current study preserved the ECM, maintain biomechanical and biocompatibility properties of the bone scaffolds. The type of bone, cortical or cancellous, site of origin, and the donor’s age also determine the chemical preparation protocol. This study employed a two week chemical processing and another seven days of an enzymatic process prior to freeze-drying. However, many other studies run their chemical processing for up to three weeks or even more [22]. Such treatment methods can potentially damage the extracellular matrix of the bone tissues making them susceptible to micro and macro fractures [23]. Similarly, thermal shock treatments which employ heating the bone samples upto 121°C followed by freezing in liquid nitrogen (-196 C) coupled with sodium bicarbonate and graded alcohol treatments for bone tissue decellularization is also reported [24]. However, the biomechanical qualities of the bone scaffolds could be jeopardized by temperature shock.

Under light microscopy, DMB scaffolds demonstrated a large number of acellular lacunae underscoring the effectiveness of HCL as a means of eliminating cells within the ECM. Acid treatment may solubilize the cell membrane and nuclear material by their intrinsically charged properties. This important role of acid treatment in bone demineralization is less highlighted in the literature because the original idea of producing DMB was focused on achieving its osteoinductive property following the release of bone-inducing proteins, named BMPs (bone morphogenic proteins) [25]. The clinical success of DMB scaffolds in multiple surgical fields, including dental surgery, neurosurgery, and orthopedic surgery [15], has been primarily attributed to their ability to release BMP at the surgical site, enhancing osteoinduction and bone regeneration. However, little was highlighted regarding the effective removal of donor cells within the ECM scaffold to avoid a chronic inflammatory host response. Although the demineralization process of the DMB scaffold in this study was able to contribute towards ECM acellularity, the findings showed that the complete elimination of cells and nuclei from the osteocyte lacunae occurred more efficiently following the decellularization process of the DCC scaffold.

Histological examination did not show the presence of adipocytes in both DMB and DCC scaffolds, suggesting the effectiveness of the defatting methods employed in the study. The Oil Red O staining further confirmed the absence of fat, which showed a 66.4% and 81% reduction of total lipids in DMB and DCC scaffolds, respectively, compared to the untreated native control bone samples. The much lower total residual lipid content in DCC scaffolds reflects the efficacy of SDS and Triton X-100 treatment in further eliminating fat from the deeper part of the scaffold, and the creation of wider pore diameter created by these detergents facilitated its effective fat clearance. Triton X-100 was applied following SDS in this study because it is a non-ionic detergent that can disrupt lipid-lipid and lipid-protein interactions, leaving protein-protein interactions intact [12]. Fat removal is critical in bone scaffold development because the presence of a layer of residual fat will prevent protein adsorption and immediate cell adhesion, a critical event occurring during the early part of scaffold implantation that determines the appropriate healing process [26]. Furthermore, residual fat in bone graft substitutes may also lead to the formation of a giant cell response resulting in chronic inflammation, fibrosis, and scarring at the implantation site [27].

SDS and non-ionic surfactant Triton X-100 debridement efforts were limited to short-duration exposure due to their inherent toxicity to tissues. Cytotoxic effects of SDS may cause damage to structural and signaling proteins [28]. Therefore, the DCC scaffolds in this study were thoroughly rinsed to eliminate the risk of toxicity and ensure the viability of reseeded cells during future implantation surgery.

In this study, deproteinization with 4% NaOCl and SDS enhanced partial bone proteins and debris removal. Higher integrity of collagen fibers in the scaffolds was preserved at such concentration instead of 6% NaOCl as advocated in other studies [29]. Cold deproteinization using a lower concentration of detergents further avoided the formation of micro-cracks on the surface of trabeculae. Micro-cracks commonly occur during the thermal deproteinization process, which causes the scaffolds to weaken due to thermal stress [30]. Heat treatment may produce cracks in the bulk material of trabeculae, thereby crucially weakening the mechanical strength of thermally deproteinized cancellous bones and making them more fragile than chemically deproteinized scaffolds. In this study, SEM analysis at 5000X magnification did not demonstrate any feature of microfracture on the surface and deeper pores of DCC scaffolds, suggesting the preservation of microstructure with the processing techniques used. This observation underscores the advantages of cold decellularization techniques employed in this study, which help maintain the architectural integrity of the bone scaffolds and preserve their intended mechanical strength for specific future functionality needs. Furthermore, maintenance of the nano-topography of the ECM is critical for the scaffold to regulate cell morphology and gene expression following the recellularization process [31]; and regulate the differentiation of stem cells into osteoblast lineage in future clinical applications [32].

The cellular remnants and DNA fragments that tend to be impacted within the deep pores in DCC scaffolds in this study were removed enzymatically using DNase and RNase [33]. Quantification of residual nucleic acids in the DCC scaffolds following enzymatic treatment in this study achieved an amount of less than 50ng/ml of dsDNA per mg of the dry weight of ECM. Furthermore, both DMB and DCC scaffolds did not show any visible DNA bands in the agarose gel. Quantification data of the residual DNA content over the different days of enzymatic treatment in DCC scaffolds further support this finding. The total RNA content was also negligible in the DCC scaffolds. The outcome of the decellularization technique in this study achieved the recommended acceptable level of residual nucleic acid content in biological tissue scaffolds for safe transplantation. [34].

Demineralization treatment of the initially defatted bovine bone blocks using 0.6M HCL effectively removed the calcium and phosphorous content while decellularized DCC scaffold maintained a large portion of calcium and almost all phosphorous content. EDS analysis in DCC scaffolds showed the typical Ca, P, and O peaks belonging to hydroxyapatite, suggesting its proximity to the physico-chemical characteristics of the mineral phase of native bone [35]. The mineral content contributed to the physical strength of the ECM, and this is reflected in the mechanical testing whereby the flexural elasticity of DCC is higher compared to DMB. Although wider and richer pores may compromise the mechanical strength of the DCC scaffold, its load-bearing ability may be protected due to the preservation of a high amount of calcium and phosphorous compared to the DMB scaffold. Bone processing techniques also modify the matrix stiffness that profoundly influences future cell growth and differentiation [30]. This study showed that the DCC scaffold demonstrated a higher matrix stiffness value than the DMB scaffold, which may later influence the survival and behavior of MSCs towards osteoblastic lineage that prefers stiffer substrate.

Preservation of an appropriate amount of residual structural protein following tissue processing would contribute to scaffold mechanical strength. The deproteinization process in this study directly affects the integrity of collagen preservation. It is difficult to evaluate the different types of deproteinization techniques applied in the xenogeneic tissue process as advocated by different authors due to the wide variation in the concentration of detergents used and duration of exposure employed, different ages of tissue donors, and diverse anatomical sites of bone scaffolds [36]. While FTIR analysis in this study demonstrated low absorbance in the spectra of amide groups related to protein presence in DMB and DCC scaffolds, the presence of collagen is still in the detectable range for both scaffolds, as confirmed by the total collagen assay. Taken together from the above, the altered ultrastructural characteristics and biochemical collagen assay and their reduced mineral content suggest that both DMB and DCC scaffolds are suitable to be used as bone fillers. At the same time, DCC may also be used as structural support in moderate load-bearing areas in future clinical applications.

In this study, the bovine bone scaffolds were lyophilized at the end of the processing stage for storage durability and ease of transportation [37]. Freeze-drying/lyophilization began in 1951 in the US Naval Tissue Banks, and it is still among the most prevalent bone graft storage methods. Lyophilization may also help reduce the antigenicity of bone grafts. Following the lyophilization process, the bovine cancellous bone scaffolds were then packed and exposed to gamma-radiation at 25 kGy, a standard dose for bone substitute sterilization [38]. Terminal radio sterilization guarantees a high-quality assurance of tissue graft, but the mechanical strength of radiated bone scaffolds may be further weakened by gamma radiation [39]. The stability of the lyophilized product at room temperature makes it suitable for clinical use despite its extra processing time needed to produce a DCC scaffold in this study seems to be an advantage [40].

The compression and three-point bending tests revealed a typical elastic-plastic deformation of engineering materials, even though each type of sample exhibited a unique deformation behavior upon the tests. The native control bone samples showed the highest elasticity and maximum strength values compared to other samples. It is indicated that the SS curve of cancellous bone has a strong dependence on the apparent bone density [26, 41]. The DMB scaffolds exhibited a typical SS curve of biopolymers with an extended low-stress flat region in the initial part of the SS curve [24, 32]. On the other hand, the SS curve of the DCC scaffolds had multiple peak stresses in the plastic region and is similar to that of a deproteinized bone [33] It is thought that DCC and deproteinized bone have the same deformation behavior because both were subjected to a leaching process using sodium hypochlorite solution even though the decellularized process uses other chemical solutions and processes.

For the three-point bending test, the native control bone samples showed the highest elasticity and maximum strength values compared to other samples. These results were consistent with the results of the compressive test. The bending properties of the DCC scaffolds were better than those of the DMB scaffolds aside from the total strain. Since the DCC scaffolds are similar to the deproteinized bone, they will be less ductile than other samples because DCC scaffolds have a trabecular architecture made of mineral phases, which have a brittle behavior. In this study, the DCC scaffolds showed an elastic-plastic behavior and behaved as a ductile material upon the compressive and bending tests, although the elasticity’s strength and modulus were smaller than the control samples. Thus, it is indicative that the DCC scaffolds have moderate biomechanical properties and are suitable for bone grafting application in dentistry.

Following the physicochemical and mechanical properties evaluations, the scaffolds were examined for their osteoconductive, osteoinductive, and osteogenesis capacity. Many metals and polymers used in biomaterial research show a high affinity for endotoxins, abundant in the environment. The presence of endotoxins may affect the evaluation of biomaterial bioactivity by inducing solid inflammatory reactions but biomaterial researchers at large have not given enough attention to the risk of endotoxin contamination [42]. This study showed that the endotoxin burden in both the DMB and DCC scaffolds is very low and meets the requirements for the FDA-acceptable level of endotoxin contamination with medical devices of 0.5 endotoxin units/ml.

The present study further explored the DMB and DCC scaffolds’ ability to support cell attachment, proliferation, and differentiation of human osteoblasts. This recellularization process begins with direct and indirect XTT assay cell proliferation studies. The findings indicated that both DMB and DCC scaffolds could support the growth of human osteoblast cells and implied that both scaffolds’ surface topography and roughness favored cell attachment and cell-cell interaction, which are imperative for the survival of cells. We observed that HOB cells attached readily to the DMB and DCC scaffolds as confirmed by DAPI staining on day 1 post-seeding. Cell attachment on the scaffold surface is favored by our method of repeated seeding and the presence of extensive porosity and interconnectivity. The repeated slow seeding of cells suspended in minimal volume employed in the present study provided maximal cell attachment/adhesion. Although not statistically significant, the DCC scaffold demonstrated greater cell proliferation than the DMB scaffold, plausibly indicating enhanced biocompatibility. Nevertheless, biologically significant changes may not always be statistically significant.

SEM showed numerous healthy osteoblasts on both scaffolds, demonstrating good attachment with their filopodia extensions. More osteoblasts were seen within the deeper pores in DCC compared to DMB scaffolds. Likely, the 3D ultrastructure, surface topology, and conserved composition of the ECM contribute to these effects [41]. Treatment protocols for both DMB and DCC scaffolds could preserve the natural pore architecture. DMB scaffolds in this study tend to have a smaller pore diameter compared to DCC scaffolds, but they are still within a good porosity range for osteoprogenitor cell adhesion and survival [43]. The wider pore diameter and extensive pore interconnectivity within the DCC scaffolds may invite richer fibrovascular invasion during the recellularization phase, providing a better nutrient supply and removing waste products for the cells. Preservation of the ultra-structure and chemical components of the ECM is critical for the osteoconductive properties of the scaffolds, allowing cells to attach, migrate, spread, and proliferate.

This study further investigated the osteoinductivity of scaffolds, which is one of the key properties of an ideal bone graft. The osteoinductivity of DMB and DCC scaffolds was assessed by evaluating the ability of seeded osteoblast cells on scaffolds to express osteogenic differentiation markers and support mineralization of the ECM in the absence of osteogenic differentiation factors. Mineralization potential was significantly higher in DCC scaffolds when compared to DMB even in the absence of osteogenic media with the release of more calcium ions, as evident from the quantification of Alizarin Red S staining. Significantly higher expressions of osteogenic markers in cells grown on DCC scaffolds, including OCN, ALP, and RUNX2, further support this finding. RUNX2 is a transcription factor essential for mesenchymal stem cells’ commitment to the osteoblast lineage, while alkaline phosphatase activity supports the propagation of osteoblast differentiation [44]. Osteocalcin is the most abundant non-collagenous protein in the bone that determines terminal differentiation and secretion of extracellular matrix ready for the mineralization process [45]. The upregulation of osteogenic genes signifies the osteoinductive capacity of the DCC scaffold in supporting bone regeneration, suggesting its inherent preserved osteogenic topographical cues.

To conclude, the present study details a decellularization strategy using the mildest processing protocol possible that yields an acellular material without disrupting the structural and functional component of its ECM. While the DMB scaffold in this study showed some degree of elimination of cellular nuclear material, the DCC scaffold demonstrated a more effective elimination of cells and genetic material, leaving a safe amount of residual nucleic acid while at the same time, partially protect and maintain its structural proteins and micro-topography, calcium, and phosphorous content. The lower immunogenicity of the DCC scaffolds may favor less risk transplantation of nucleic acid into the future recipients. In addition, maintenance of collagen in DCC help preserve the presence of the binding site and enhancing the biocompatibility of the scaffolds beside providing advantageous biomechanical properties. The DCC scaffolds also demonstrated wider pores with extensive interconnectivity. These superior physico-chemical characteristics allowed the DCC scaffold to support a higher osteoblast cell proliferation rate, favoring the upregulation of osteogenic genes and producing abundant mineralization nodules *in-vitro*. Under the current experimental conditions, our DCC scaffold favorably supports the mechanisms of osteoconduction, osteoinduction, and osteogenesis as a promising biocompatible and plausible immuno-compatible bone graft substitute; yet to be examined in future *in-vivo* studies.

In addition, preservation of calcium and phosphate also gave the DCC scaffold a higher mechanical strength and stiffness compared to DMB. This study underscores the efficacy of naturally mineralized scaffolds in supporting bone regeneration and demonstrated the differences in outcome performance of mineralized scaffolds when processed in two different ways [46, 47]. It highlighted the challenge in disposing of native cells to eliminate antigenicity while attempting to preserve natural ECM properties. There is evidence, however, that native cell counts alone do not correlate with the residual antigen content of a tissue [35]. Therefore, there is a need to perform further biological studies such as *in-vitro* peripheral blood monocyte response tests to assess the immuno-compatibility of DCC scaffolds as a bovine bone substitute for tissue engineering applications in the near future.

## Supporting information

S1 Raw images (PDF)
