# Correction: Evaluation of decellularization process for developing osteogenic bovine cancellous bone scaffolds *in-vitro*

**DOI:** 10.1371/journal.pone.0307687

**Published:** 2024-07-18

**Authors:** The PLOS ONE Staff

There are errors in the Author Contributions. The correct contributions are:

**Conceptualization:** Ali Al Qabbani, Sausan AlKawas, Suzina Sheikh Abdul Hamid, A. R. Samsudin, Ahmad Azlina.

**Data curation:** Junaidi Syarif, Suzina Sheikh Abdul Hamid, Ahmad Azlina.

**Formal analysis:** Ali Al Qabbani.

**Investigation:** Ali Al Qabbani, K. G. Aghila Rani, Sausan AlKawas, A. R. Samsudin.

**Methodology:** Ali Al Qabbani, K. G. Aghila Rani, Junaidi Syarif, Sausan AlKawas, Ahmad Azlina.

**Resources:** K. G. Aghila Rani, Junaidi Syarif, A. R. Samsudin.

**Supervision:** Suzina Sheikh Abdul Hamid, A. R. Samsudin, Ahmad Azlina.

**Validation:** K. G. Aghila Rani, Junaidi Syarif, Sausan AlKawas.

**Writing–original draft:** Ali Al Qabbani, A. R. Samsudin.

**Writing–review & editing:** Ali Al Qabbani, K. G. Aghila Rani, Junaidi Syarif, Sausan AlKawas, Suzina Sheikh, Abdul Hamid, A. R. Samsudin, Ahmad Azlina.

The affiliation for the second author is incorrect. The correct affiliation is not indicated. K. G. Aghila Rani is not affiliated with #3 but with: Research Institute of Medical and Health Sciences, University of Sharjah, Sharjah, United Arab Emirates.

The publisher apologizes for the errors.
