# Supplementary material for: Evaluation of decellularization process for developing osteogenic bovine cancellous bone scaffolds in-vitro
Source: PLoS One. 2023 Apr 5;18(4):e0283922. doi: 10.1371/journal.pone.0283922 (PMC10075422; doi:10.1371/journal.pone.0283922)
Supplement: S1 Raw images — (PDF) [file pone.0283922.s001.pdf]

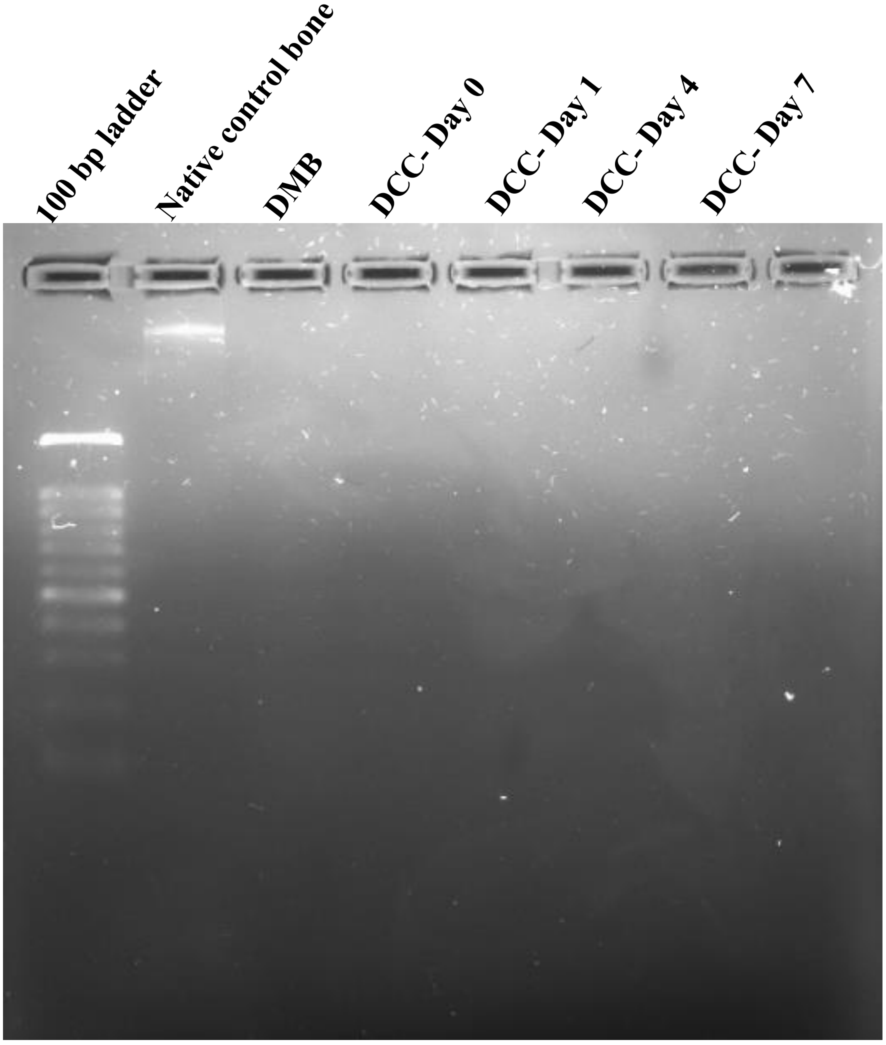

S1_raw_image: Agarose gel electrophoresis image of the total DNA extracted from DMB scaffold, DCC without exposure to DNase/RNase (Day 0), and on days 1, 4, and 7 post-DNase treatment of DCC scaffold compared to native control bone. The image was captured using a ChemiDoc Touch Imaging System (Bio-Rad, USA). This original gel image is used in Figure 6c.
